# MicroRNA profiling of mouse liver in response to DENV-1 infection by deep sequencing

**DOI:** 10.7717/peerj.6697

**Published:** 2019-04-22

**Authors:** Lian Yih Pong, Sinikka Parkkinen, Amreeta Dhanoa, Han Ming Gan, Indeevari Abisheka Chiharu Wickremesinghe, Sharifah Syed Hassan

**Affiliations:** 1 Jeffrey Cheah School of Medicine and Health Sciences, Monash University Malaysia, Bandar Sunway, Selangor, Malaysia; 2 Infectious Diseases and Health Cluster, Tropical Medicine and Biology Platform, Monash University Malaysia, Bandar Sunway, Selangor, Malaysia; 3 Department of Biology, University of Eastern Finland, Joensuu, North Karelia, Finland; 4 School of Science, Monash University Malaysia, Bandar Sunway, Selangor, Malaysia; 5 Centre for Integrative Ecology, School of Life and Environmental Sciences, Deakin University, Geelong, Victoria, Australia

**Keywords:** Mouse liver, MiSeq sequencing, Intracellular miRNA, Dengue virus, Host-virus interaction, Small RNA

## Abstract

**Background:**

Dengue caused by dengue virus (DENV) serotypes −1 to −4 is the most important mosquito-borne viral disease in the tropical and sub-tropical countries worldwide. Yet many of the pathophysiological mechanisms of host responses during DENV infection remain largely unknown and incompletely understood.

**Methods:**

Using a mouse model, the miRNA expressions in liver during DENV-1 infection was investigated using high throughput miRNA sequencing. The differential expressions of miRNAs were then validated by qPCR, followed by target genes prediction. The identified miRNA targets were subjected to gene ontology (GO) annotation and pathway enrichment analysis to elucidate the potential biological pathways and molecular mechanisms associated with DENV-1 infection.

**Results:**

A total of 224 and 372 miRNAs out of 433 known mouse miRNAs were detected in the livers of DENV-1-infected and uninfected mice, respectively; of these, 207 miRNAs were present in both libraries. The miR-148a-3p and miR-122-5p were the two most abundant miRNAs in both groups. Thirty-one miRNAs were found to have at least 2-fold change in upregulation or downregulation, in which seven miRNAs were upregulated and 24 miRNAs were downregulated in the DENV-1-infected mouse livers. The miR-1a-3p was found to be the most downregulated miRNA in the DENV-1-infected mouse livers, with a significant fold change of 0.10. To validate the miRNA sequencing result, the expression pattern of 12 miRNAs, which were highly differentially expressed or most abundant, were assessed by qPCR and nine of them correlated positively with the one observed in deep sequencing. *In silico* functional analysis revealed that the adaptive immune responses involving TGF-beta, MAPK, PI3K-Akt, Rap1, Wnt and Ras signalling pathways were modulated collectively by 23 highly differentially expressed miRNAs during DENV-1 infection.

**Conclusion:**

This study provides the first insight into the global miRNA expressions of mouse livers in response to DENV-1 infection *in vivo* and the possible roles of miRNAs in modulating the adaptive immune responses during DENV-1 infection.

## Introduction

Dengue virus (DENV) is a positive-sense single-stranded, mosquito-borne RNA virus that belongs to the genus *Flavivirus* of the *Flaviviridae* family. DENV infections are caused by at least four distinct serotypes of DENV (DENV-1, DENV-2, DENV-3 and DENV-4). The majority of primary dengue infections with any one of the DENV serotypes results in a mild, self-limiting flu-like illness known as dengue fever (DF). The majority of DF patients recover without intervention, although a 2–5% develop more severe manifestations ranging from dengue hemorrhagic fever (DHF) to dengue shock syndrome (DSS) (Murrell, Wu & Butler, 2011; Halstead, 2002). Infection with one serotype confers life-long immunity to that serotype; however, it does not protect the host against infections with other serotypes (Mukhopadhyay, Kuhn & Rossmann, 2005). Liver impairment has been extensively reported in acute dengue infection of human. Severe dengue can lead to liver failure which can be complicated by encephalopathy, severe bleeding, renal failure, metabolic acidosis and fatal outcomes (Green & Rothman, 2006; Halstead, 2007; Aye et al., 2014; Samanta & Sharma, 2015; Fernando et al., 2016). As the liver is one of the main organs involved in dengue infections, the underlying pathways involved in causation of liver damage in primary DENV infection need to be unraveled as this remains poorly understood.

MicroRNA (miRNA) profiling has been widely used to gain deeper understanding of the molecular mechanisms of host-pathogen interactions, especially involving host miRNAs modulation of gene expressions and their levels in response to a disease. miRNAs are small, endogenous non-coding single-stranded RNA that are approximately 22 nucleotides in size. miRNAs regulate the post-transcriptional of gene expression in animals and plants by base-pairing with the complementary sequences within 3′ non-translated region (NTR) of the targeted mRNA, thus inducing gene silencing via repression of protein synthesis or degradation of mRNA targets (Bartel, 2004; Fabian, Sonenberg & Filipowicz, 2010).

The differential expressions of intracellular miRNAs in mosquitoes either *in vivo* or *in vitro*, and peripheral blood mononuclear cells (PBMC) in response to DENV-2 infection have been profiled; findings suggest that the host miRNAs were modulated by regulation of the immune-related genes during the antiviral responses against dengue virus (Qi et al., 2013; Campbell et al., 2014; Liu et al., 2015; Liu et al., 2016; Miesen et al., 2016). However, the miRNA profiling or the involvement of intracellular miRNAs in the pathophysiological mechanisms of dengue infection in a mammalian model remain unreported. In this study, BALB/c mouse strain was used to study the miRNA expression in mouse liver in response to DENV infection. Although there is no non-human species that naturally develop dengue disease similar to that observed in humans, the immunocompetent mouse models including BALB/c mouse strain have been shown to be permissive to DENV infection and replication (Huang et al., 2000; Chen et al., 2004; Paes et al., 2005; Bente & Rico-Hesse, 2006; Paes et al., 2009; Tuiskunen et al., 2011). Moreover, the liver injury associated with DENV infection has been evidenced in BALB/c mouse strain (Paes et al., 2005; Paes et al., 2009; França, Zucoloto & Da Fonseca, 2010; Tuiskunen et al., 2011; Sakinah et al., 2017).

In this study, we have explored the miRNA dysregulation in liver of mice with DENV-1 infection by using deep sequencing. We aimed to utilize the differential expression of intracellular miRNAs to examine the pathophysiological mechanisms of host responses during DENV-1 infection. We then performed a comparative analysis on the differential expression of miRNAs followed by the prediction of genes targeted by the high differentially expressed miRNAs. The identified miRNA targets were subjected to gene ontology (GO) annotation and pathway enrichment analysis to elucidate the potential biological pathways and molecular mechanisms that are modulated during DENV-1 infection.

## Materials & Methods

### Dengue viruses and cell culture

Dengue virus serotype 1 (DENV-1) used in this study was isolated from human serum diagnosed with dengue, which was a generous gift from Professor Sazaly Abu Bakar of Tropical Infectious Diseases Research and Education Centre (TIDREC), University of Malaya, Malaysia (GenBank accession number FR666924.1). Following initial passages in C6/36 cells (*Aedes albopictus* mosquito cells; ATCC CRL-1660), DENV-1 was propagated in Vero cells (African green monkey kidney cells; ATCC CCL-81) in minimum essential media (MEM) supplemented with 2% FBS, 1% HEPES and 1% penicillin-streptomycin antibiotic (Gibco) to obtain high viral titer. The virus stocks were stored at −80 °C until further use. Viral stocks were concentrated by ultracentrifugation at 30,000 rpm for 3 h at 4 °C and resuspended in MEM. The concentrated DENV-1 was titrated via TCID_50_ assay and the viral titer was determined using the Spearman-Karber method (Hierholzer & Killington, 1996).

### DENV-1 infection in mice

Evidence that DENV-1 infection was established in the mice was based on the development of IgM and IgG antibodies at various days post infection to determine the peak levels of IgM and IgG (Wickremsinghe et al., 2018). Three six-week old male BALB/c mice were infected with DENV-1. Briefly, each mouse was inoculated with 200 µL of MEM containing 1.26 × 10^7^ TCID_50_/mL DENV-1: a total of 100 µL were administered intravenously and the other 100 µL was administered subcutaneously into each mouse. Three aged-matched BALB/c mice without DENV-1 infection were used as control. Mice were euthanized 3 days post infection (d.p.i) and the liver of each mouse was collected and stored at –152 °C. During the three days of observation after infection with DENV-1, all mice survived the infection without apparent signs of the disease and did not show any signs of distress. On post mortem, it was observed that the spleens of infected mice were grossly enlarged and the livers of infected mice were slightly enlarged when compared to mock-infected mice (data not shown). The animal experiment was approved by Monash University Animal Ethics Committee (Approval No. MARP/2015/071). The mice were housed in individually ventilated mice cages, which is also mosquito proof.

### RNA isolation and library preparation

The small RNA (sRNA) of each sample was isolated from a pool of three mouse livers by using mirVana miRNA Isolation kit (Ambion) according to the manufacturer’s protocol. A total of 100 ng of enriched sRNA from each sample was used to construct a respective library by using the NEBNext^®^ Small RNA Library Prep Set for Illumina^®^ (NEB) according to the manufacturer’s instruction. Each library was constructed with a unique index adaptor barcode in order to enable pooled multiplex sequencing. Briefly, the adaptor-ligated sRNA was reverse-transcribed into cDNA followed by PCR amplification for 15 cycles. The sRNA libraries were then size-selected using AMpure XP Beads (Beckman Coulter) to recover the library DNA with 136–143 bp in length which was the average size of sRNA with 3′and 5′adaptors attached. The quantity and size distribution of the libraries was analyzed by Agilent 2100 Bioanalyzer (Agilent Technologies) using High Sensitivity DNA assay.

### Deep sequencing and bioinformatics analysis

A total of two libraries were normalized to 2 nM and pooled for sequencing on the Illumina MiSeq Benchtop Sequencer at Monash University Malaysia Genomics Facility (1 ×36 bp configuration). The post sequencing data processing was carried out using the UEA sRNA Workbench (Stocks et al., 2012). The adaptor sequences were trimmed and removed from the raw reads according to the 3′ adapter sequence (AGATCGGAAGAGCACACGTCT) obtained from the NEBNext^®^ Small RNA Library Prep Set for Illumina^®^ (NEB) instruction manual using the first eight nucleotides. The adapter-removed reads were then filtered to the length between 16 bp and 35 bp followed by filtering against low complexity sequences which contain less than three distinct nucleotides. The reads matching to known transfer and ribosomal RNA sequences were excluded using the database from RFAM, v10 (Burge et al., 2013). Finally, the clean reads were converted into FASTA format and then were mapped to the *Mus musculus* miRNA dataset from miRBase (version 20) using miRProf tool from UEA sRNA Workbench (Kozomara & Griffiths-Jones, 2011; Kozomara & Griffiths-Jones, 2014). Parameters were set to allow overhangs, only kept best matches, and disallowed variant or same miRNA family groupings. The miRNA sequencing data are available from the NCBI Gene Expression Omnibus (GEO) and is accessible through accession number GSE123346.

### Analysis of differential miRNA expression

To compare the miRNA abundance between two sRNA libraries, the mappable reads were normalized using transcripts per million (TPM) (Liu et al., 2015; Jiang & Sun, 2018). TPM was calculated where the absolute number of read of an individual miRNA in a particular sRNA library was divided by the absolute total number of read of all miRNAs in this library and then multiplied by one million. The miRNAs with TPM value less than 10 in both libraries were excluded from differential expression analysis (Liu et al., 2015). The miRNAs with changes in expression of a log2-fold change higher than 1 (upregulation) or lower than −1 (downregulation) between DENV-1 infection and uninfected control were identified as highly differentially expressed miRNAs.

### Quantitative real-time PCR (qPCR)

Some of the differentially expressed and most abundant miRNAs were selected for validation by two-step qPCR. The qPCR validation was performed in StepOnePlus Real-Time PCR System (ABI) by using TaqMan microRNA Assay (ABI) according to manufacturer’s instructions. Each of the samples was assayed by qPCR in triplicate. The snoRNA202 was used as an endogenous control for miRNA in this qPCR assay. The resulted qPCR data were analyzed using StepOne Software v2.3 (ABI). The relative expression of miRNAs was statistically calculated by using Relative Expression Software Tool (REST) and the resulted relative expression ratio (R) was tested for significance by a Pair Wise Fixed Reallocation Randomization Test which is included in REST (Pfaffl, Horgan & Dempfle, 2002).

### miRNA target prediction, GO and pathway enrichment analyses

The target genes of all highly differentially expressed miRNAs were predicted and identified using two web-based databases of mouse species: the microT-CDS (version 5.0) and TarBase v7.0 (version 7.0) in Diana Tools (Paraskevopoulou et al., 2013; Vlachos et al., 2015a). microT-CDS was used with default parameters (microT threshold: 0.8; *p*-value threshold: 0.05), while *p*-value threshold in TarBase v7.0, which determines the miRNA targets based on the experimental data, was set to 0.05. The miRNA target genes were then annotated through GO term enrichment and pathway analysis using mirPath v3.0 in Diana Tools (http://www.microrna.gr/miRPathv3/) (Vlachos et al., 2015b). The threshold of EASE score, a modified Fisher Exact *p*-value was set to 0.05 to sort out the terms where genes are considered strongly enriched in the annotation. GO terms and pathways with a *p*-value less than 0.05 were defined as statistically significant.

## Results

### Analysis of small RNA libraries by high-throughput sequencing

**Figure 1:**
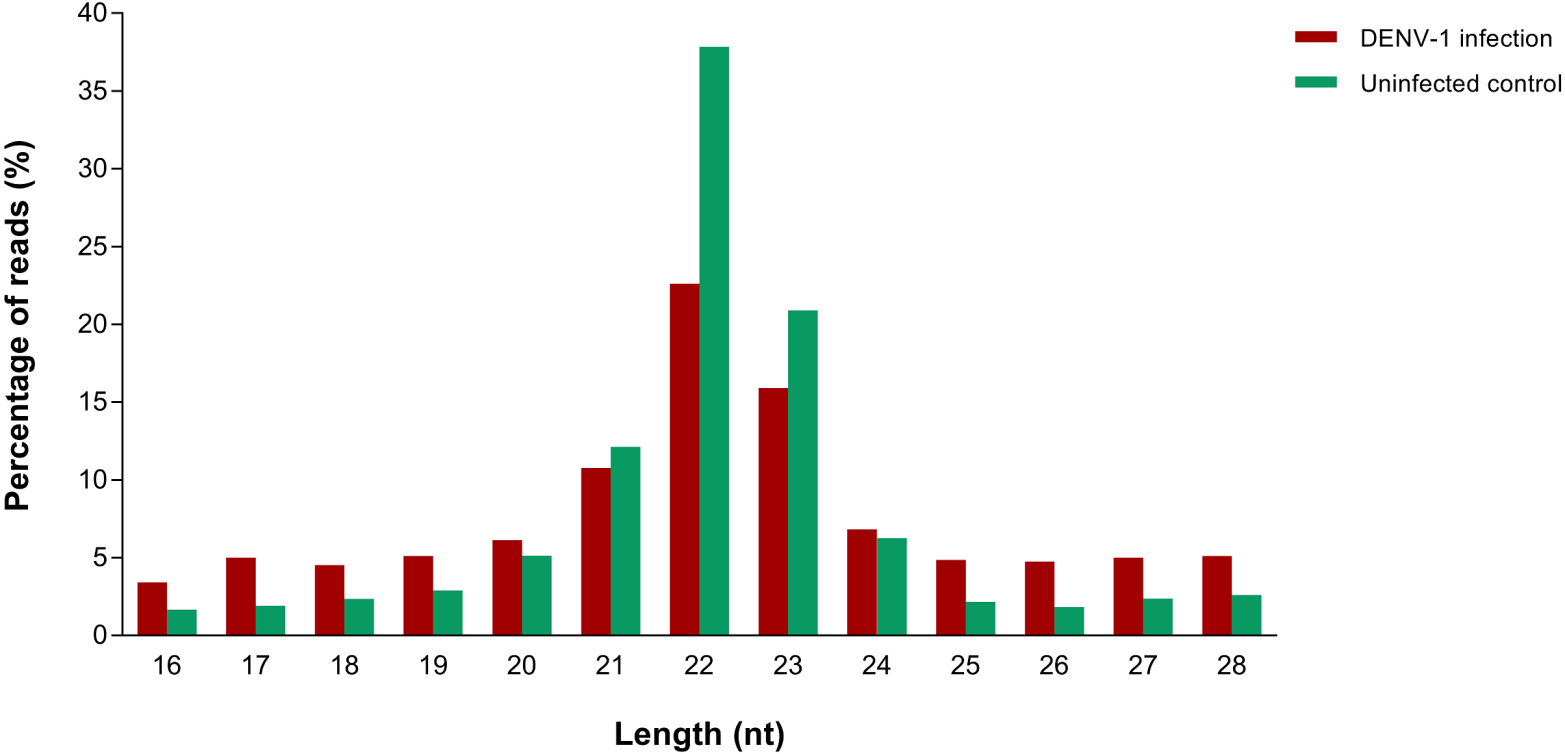
Read size distribution of clean reads from deep sequencing in DENV-1-infected and uninfected mouse livers.

A total of 4,753,961 and 2,229,568 raw reads were generated from the small RNA libraries of uninfected control and primary infection, respectively. After the adaptor removal and multiple filtering steps as described in the methodology, 572,866 out of 1,316,491 clean reads from uninfected control library and 133,348 out of 527,208 clean reads from DENV-1 infection library were mapped to the *Mus musculus* miRNA dataset from miRBase (version 20). The size distribution of clean reads was similar in both libraries and most of the reads were 22 nucleotides in size (Fig. 1).

### Detection of miRNAs and its abundance in livers of DENV-1-infected and uninfected mice

**Figure 2:**
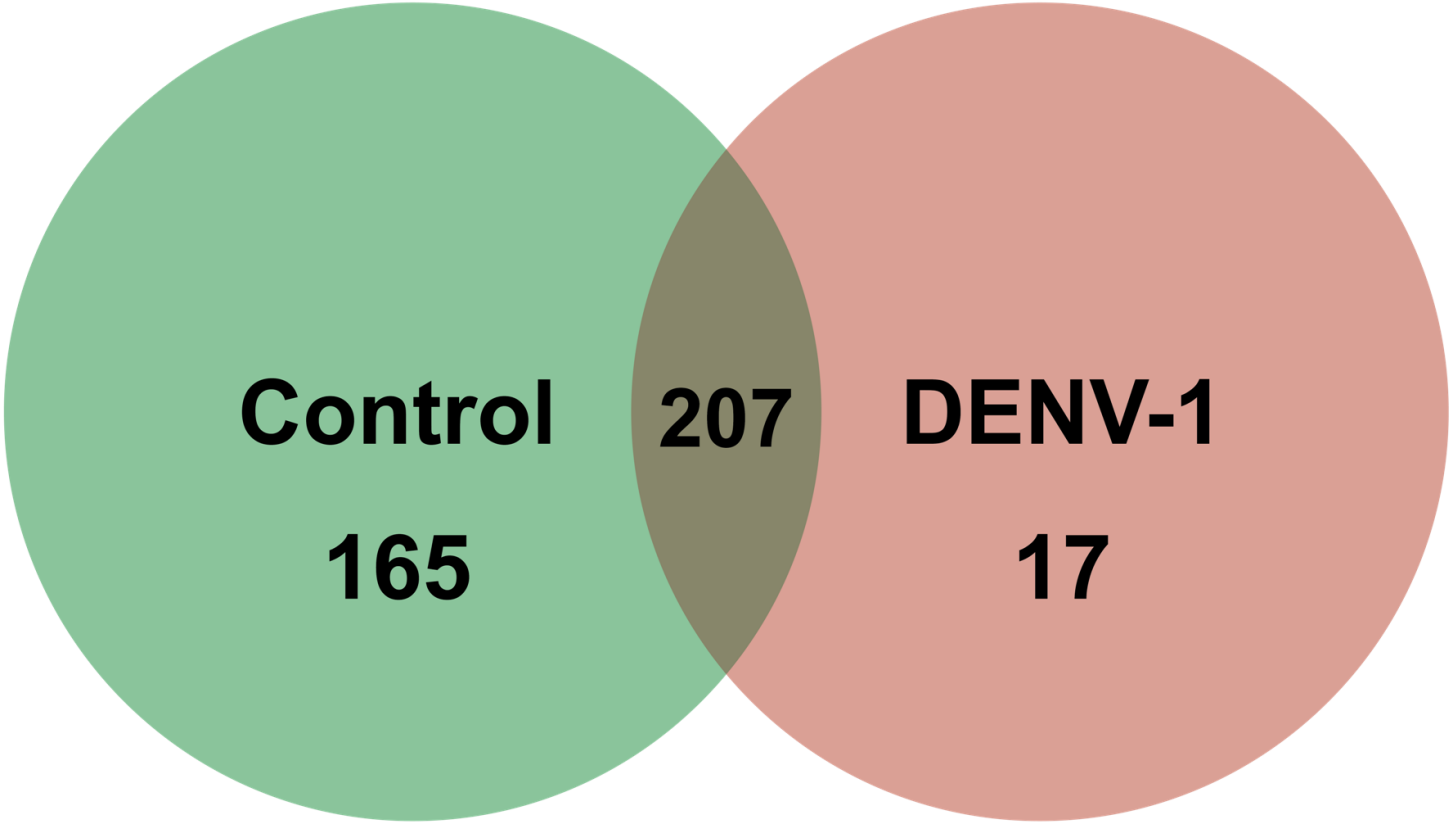
Venn chart depicting the number of miRNAs detected in the DENV-1-infected and uninfected mouse livers. Detection of these miRNAs was done by mapping their clean reads to the *Mus musculus* miRNA dataset from miRBase (version 20).

**Figure 3:**
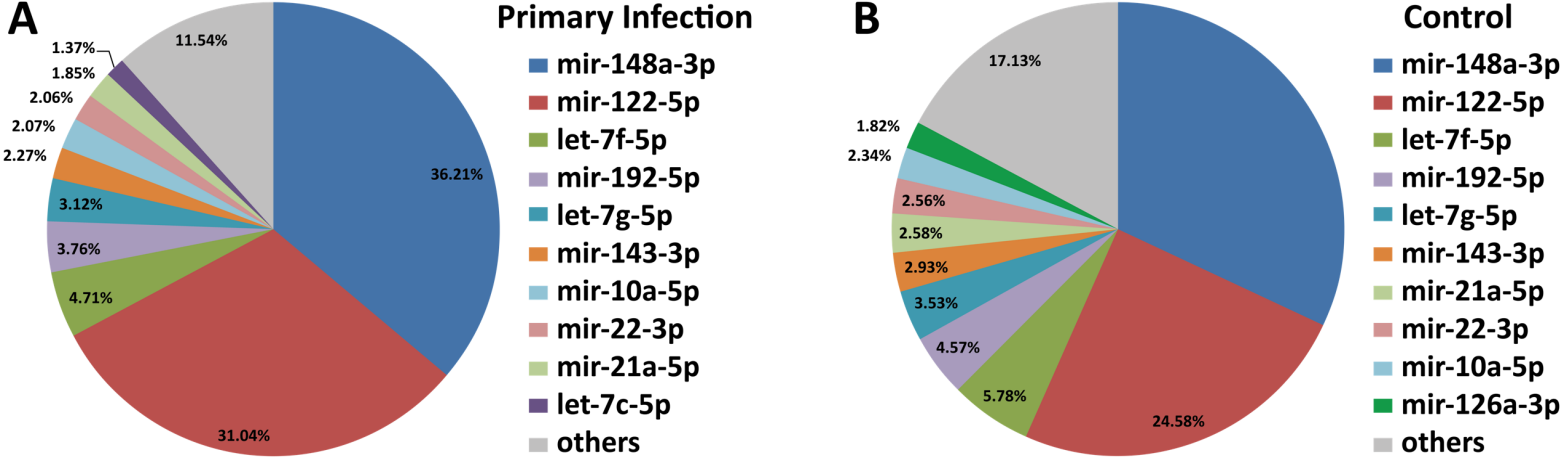
Distribution of the top 10 most abundant miRNAs detected in DENV-1-infected and uninfected mouse livers. (A) Library of DENV-1-infected mouse livers. (B) Library of uninfected control mouse livers. The others category represents all remaining miRNAs in the mouse miRNA dataset from miRBase (version 20).

Out of 433 known mouse miRNAs, 372 miRNAs in livers of uninfected mice library and 224 miRNAs in livers of DENV-1-infected mice library were detected regardless of the TPM value of less than 10 in both libraries (Data S1). Of these, 207 miRNAs were found in both libraries. A total of 17 different miRNAs were only found in DENV-1-infected mice livers in comparison with the uninfected control (Fig. 2). Forty-four miRNAs were not detected in both libraries (Data S2). There were 10 most abundant miRNAs that made up more than 80% of the total mappable reads across each of the libraries (Fig. 3). All of these 10 miRNAs were observed in both libraries except let-7c-5p and miR-126a-3p. The miR-148a-3p and miR-122-5p were found to be the two most abundant miRNAs in each of the libraries, covering at least 50% of the respective total mappable reads.

### Differential miRNA expression between livers of DENV-1-infected and uninfected mice

**Figure 4:**
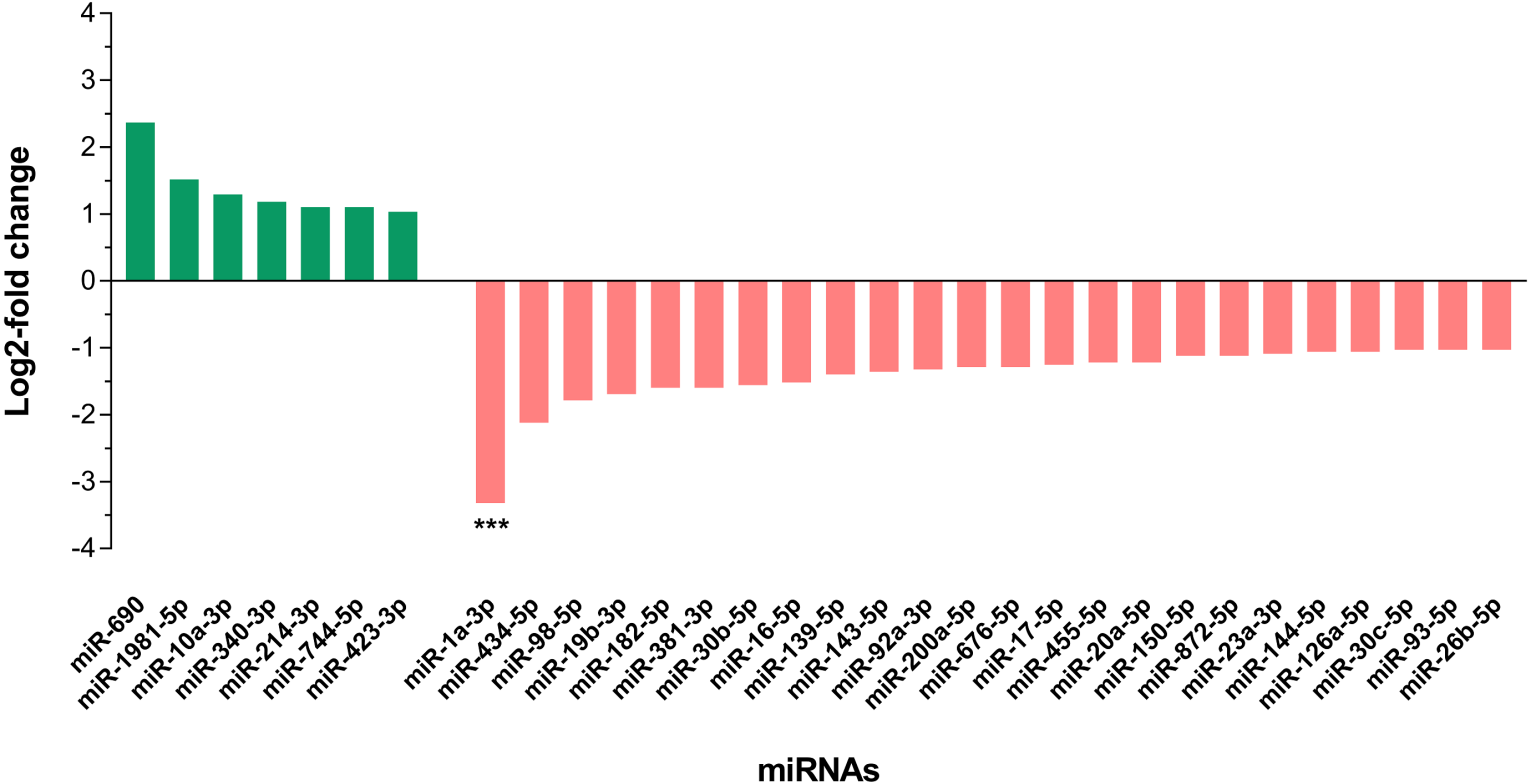
Differential expression of miRNAs in the livers of DENV-1-infected and uninfected mice. Bar graph depicts the highly differentially expressed miRNAs, in which seven miRNAs were upregulated and 24 miRNAs were downregulated by at least 2-fold change in the livers of DENV-1-infected mice relative to uninfected control. The data was subjected to statistical analysis using ordinary two–way ANOVA followed by Sidak’s multiple comparison test. *** *p* = 0.0001.

In differential expression analysis, a total of 155 miRNAs with TPM value more than 10 in both libraries were retained for further analysis (Campbell et al., 2014; Liu et al., 2015) (Data S3). Of these, 31 miRNAs were found to have at least 2-fold change in upregulation or downregulation regardless of miRNA abundance (Data S3); seven miRNAs were upregulated and the highest fold change of 5.16 was observed in miR-690, whilst 24 miRNAs were downregulated in the livers of DENV-1-infected mice in comparison to uninfected control (Fig. 4). The miR-1a-3p was the most downregulated miRNA in DENV-1-infected mouse livers, with a significant fold change of 0.10. In addition, twenty-eight miRNAs were not detected in the livers of DENV-1-infected mice but were only detected in uninfected control with TPM value of more than 10, which were let-7d-3p, miR-133a-3p, miR-133b-3p, miR-152-5p, miR-15a-5p, miR-15b-3p, miR-181a-1-3p, miR-184-3p, miR-192-3p, miR-193a-3p, miR-199b-5p, miR-19a-3p, miR-205-5p, miR-206-3p, miR-217-5p, miR-26b-3p, miR-29c-5p, miR-300-3p, miR-30b-3p, miR-342-3p, miR-375-3p, miR-378b, miR-434-3p, miR-501-3p, miR-5099, miR-574-3p, miR-7a-1-3p and mir-802-3p (Data S4). It is deemed that these miRNAs were downregulated during dengue infection with respect to the uninfected control. miR-339-3p was detected in the livers of DENV-1-infected mice with TPM value of more than 10 but it was not detected in the uninfected control (Data S4).

### Validation of miRNAs differential expression by qPCR

**Figure 5:**
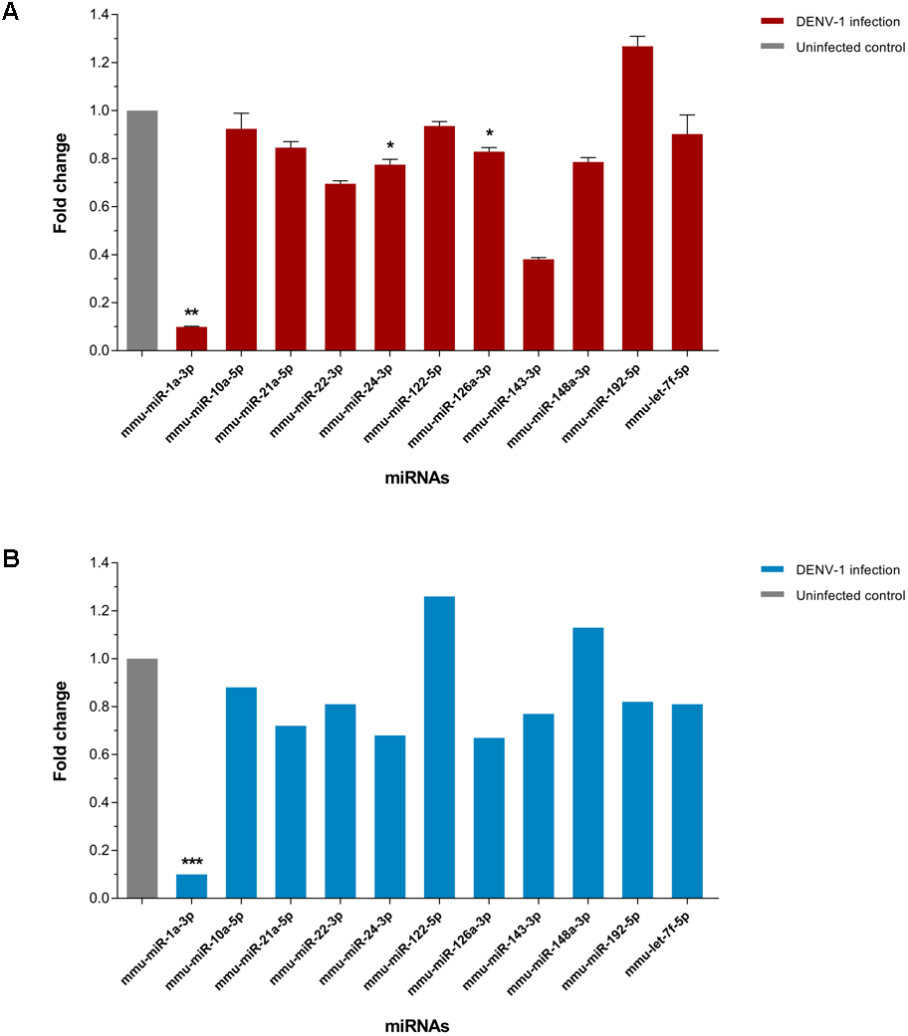
qPCR validation of miRNA expression in the livers of DENV-1-infected mice. (A) A total of 11 miRNAs expression were analyzed via two-step qPCR by using TaqMan microRNA Assay (ABI), where each of the samples was assayed in triplicate. The qPCR data was analyzed using StepOne Software v2.3 (ABI) followed by statistical analysis using Relative Expression Software Tool (REST). Bar graph depicts the fold change of miRNA expression in the livers of DENV-1-infected mice with respect to uninfected control. The vertical bar represents the standard error of mean (*n* = 3). *p* < 0.05, **p = 0.001. (B) The fold change of differentially expressed miRNAs observed in deep sequencing, which were validated by qPCR as shown in (A). The data was subjected to statistical analysis using ordinary twoway ANOVA followed by Sidak’s multiple comparison test. ***p = 0.0001.

The expression of few differentially expressed miRNAs including the most downregulated miRNA, miR-1a-3p and some of the most abundant miRNAs were validated by two-step qPCR. The qPCR result showed that expression of all 11 miRNAs except miR-122-5p, miR-148a-3p and miR-192-5p in the livers of DENV-1-infected mice have a positive correlation in expression pattern with the one observed by deep sequencing (Fig. 5). The expression of miR-1a-3p was significantly down-regulated by 10.1-fold relative to its expression in uninfected control. The other two miRNAs, miR-24-3p and miR-126a-3p were significantly downregulated by 1.3-fold and 1.2-fold with respect to uninfected control, respectively.

### miRNA target gene prediction and enrichment analyses of GO and pathways

The GO terms and pathways that are associated with 31 highly differentially expressed miRNAs were analyzed using mirPath v3.0 in Diana Tools (Vlachos et al., 2015b). In this web-based software, the predicted target genes of those 31 miRNAs were identified by using two web-based databases of mouse species, the microT-CDS (version 5.0) and TarBase v7.0 (version 7.0) in Diana Tools (Paraskevopoulou et al., 2013; Vlachos et al., 2015a). The microT-CDS is a miRNA target prediction tool, while TarBase v7.0 determines the miRNA targets based on the experimental data.

The predicted genes, which were regulated by the highly differentially expressed miRNAs, were found to be associated significantly with the biological processes, molecular functions and cell components as summarized in Table 1. These GO terms also associated significantly with the miRNA targets that are experimentally supported. The anatomical structure development and embryo development are the biological processes that involve cell differentiation. The biological processes of cell differentiation, cell division, cell cycle and cell death are involved in cell proliferation and apoptotic process. These two biological pathways, cell proliferation and apoptotic process together with the inflammatory response, which also involves cell proliferation and cell death, are part of the adaptive immune response. In addition, the inflammatory response and apoptotic process, which both involve cell death, as well as the cell differentiation are part of the innate immune response. Hence, those GO terms that are enriched significantly by the target genes are closely related to the immune responses.

**Table 1:** GO terms of target genes of 31 highly differentially expressed miRNAs in DENV-1-infected mouse liver.

**Ontology and Accession**	**Term**	**Gene Count**	***p*-Value**^a^
**Biological Process**			
GO:0048856	Anatomical structure development	1,372	1.04E–256
GO:0030154	Cell differentiation	1,040	7.49E–150
GO:0009790	Embryo development	420	2.50E–97
GO:0000902	Cell morphogenesis	315	1.79E–60
GO:0048646	Anatomical structure formation involved in morphogenesis	333	5.29E–56
GO:0006464	Cellular protein modification process	750	3.98E–38
GO:0051276	Chromosome organization	209	1.42E–27
GO:0034641	Cellular nitrogen compound metabolic process	1,249	4.32E–24
GO:0009058	Biosynthetic process	1,094	7.81E–21
GO:0048870	Cell motility	226	3.82E–20
GO:0040007	Growth	169	1.81E–17
GO:0021700	Developmental maturation	73	9.69E–17
GO:0007010	Cytoskeleton organization	246	1.54E–14
GO:0051301	Cell division	178	3.04E–13
GO:0042592	Homeostatic process	263	8.15E–10
GO:0007049	Cell cycle	305	2.27E–07
GO:0003013	Circulatory system process	58	5.46E–06
GO:0022607	Cellular component assembly	346	5.49E–06
GO:0008219	Cell death	253	2.39E–05
GO:0001701	In utero embryonic development	122	3.08E–04
GO:0007267	Cell–cell signaling^b^	179	1.07E–03
GO:0034330	Cell junction organization	44	0.031
GO:0042475	Odontogenesis of dentin-containing tooth^b^	35	0.039
GO:0045893	Positive regulation of transcription, DNA-templated^b^	273	0.046
**Molecular Function**			
GO:0043167	Ion binding	1,849	8.46E–73
GO:0001071	Nucleic acid binding transcription factor activity	360	6.64E–29
GO:0000988	Protein binding transcription factor activity	146	3.36E–08
GO:0008092	Cytoskeletal protein binding	224	9.92E–07
GO:0001077	RNA polymerase II core promoter proximal region sequence-specific DNA binding transcription factor activity involved in positive regulation of transcription ^b^	95	6.45E–03
GO:0030234	Enzyme regulator activity	210	0.043
**Cell Component**			
GO:0005623	Cell	3,929	0
GO:0005622	Intracellular	3,428	0
GO:0043226	Organelle	2,858	3.34E–94
GO:0005856	Cytoskeleton	477	1.03E–12
GO:0016023	Cytoplasmic membrane-bounded vesicle	168	6.62E–08
GO:0000228	Nuclear chromosome	79	2.96E–07
GO:0043234	Protein complex	1,015	1.10E–06
GO:0005737	Cytoplasm	2,627	9.14E–03
GO:0005768	Endosome	194	0.012
GO:0005694	Chromosome	178	0.016

**Notes.**

a GO terms with a *p*-value lower than 0.05 were defined as statistically significant.

b GO terms that were not found when analyzed using TarBase v7.0 (database of experimentally supported miRNA targets).

**Figure 6:**
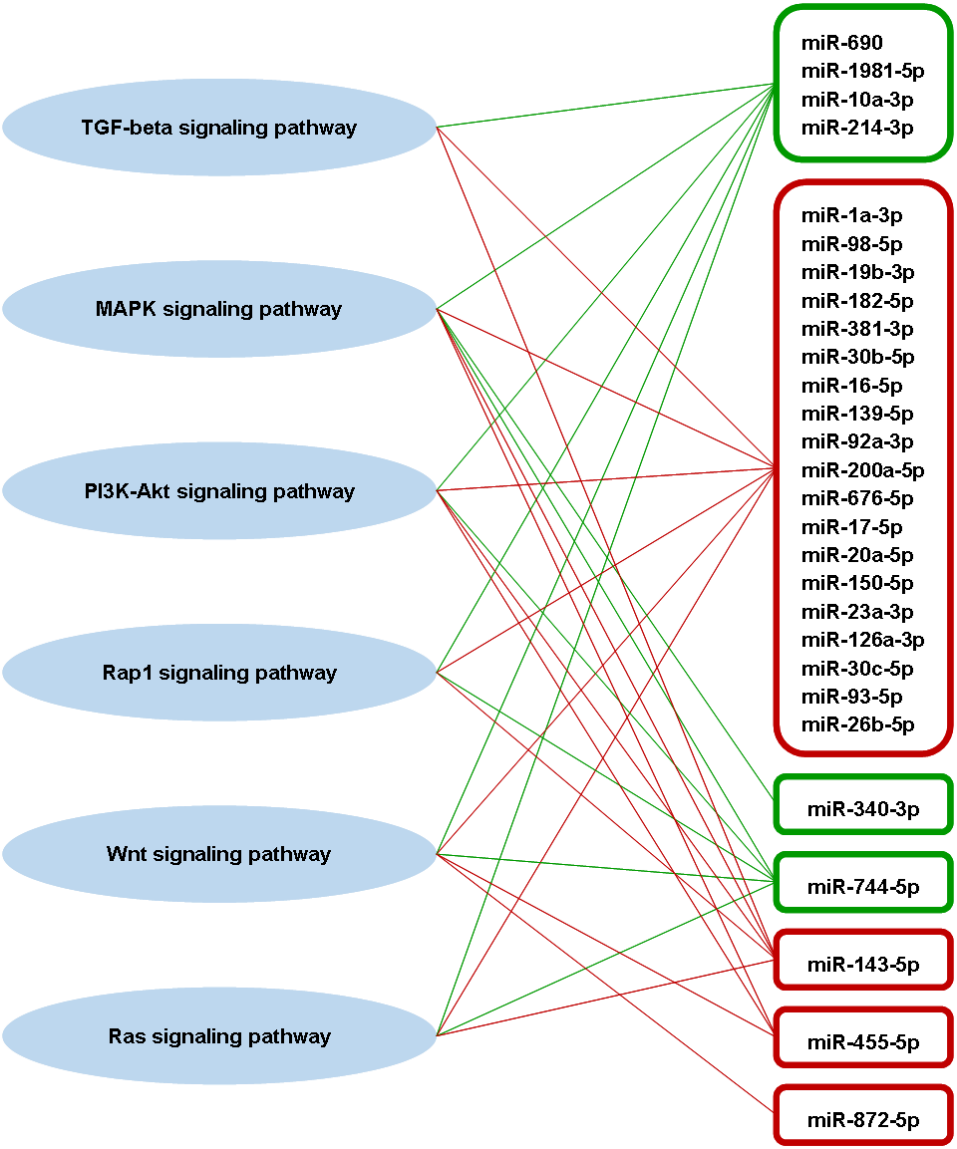
Putative interactions between the highly differentially expressed miRNAs and pathways involved in adaptive immune responses. The downregulated and upregulated miRNAs are shown in red-lined and green-lined rounded rectangle, respectively. Out of 31 highly differentially expressed miRNAs, only three miRNAs were not associated with these immune-related pathways, which were miR-423-3p, miR-434-5p and miR-144-5p.

A total of 63 pathways that are defined by the Kyoto Encyclopaedia of Genes and Genomes (KEGG) were significantly enriched by the predicted genes of part of the highly differentially expressed miRNAs (Table S1). Among these pathways, four of them are involved in adaptive immune responses, which are transforming growth factor-beta (TGF-beta) signaling pathway, mitogen-activated protein kinase (MAPK) signaling pathway, phosphatidylinositol 3′-kinase (PI3K)-Akt signaling pathway, and Rap1 signaling pathway (Katagiri et al., 2002; Zhang & Dong, 2005; Yan, Liu & Chen, 2009; Okkenhaug, Turner & Gold, 2014). These four immune-related pathways also associated significantly with the miRNA targets that are experimentally supported. Besides that, Wnt and Ras signaling pathways, which are also involved in adaptive immune response (Lapinski & King, 2012; Swafford & Manicassamy, 2015), were significantly enriched by the predicted targets of some high differentially expressed miRNAs. A total of 23 out of 31 high differentially expressed miRNAs were found associated significantly in those six immune-related pathways (Fig. 6). There were three high differentially expressed miRNAs not associated with those pathways, which were miR-423-3p, miR-434-5p and miR-144-5p. These pathways were mostly associated with the downregulated miRNAs. As the miRNA causes gene silencing via translational repression or mRNA degradation, it is likely that these pathways are activated during DENV-1 infection due to the low level of regulating miRNAs.

## Discussion

In this study, we have infected BALB/c mice with DENV-1 and the expression of miRNAs in mice livers were investigated by deep sequencing. The liver was analyzed at 3 days post DENV-1 infection, that is, at an early stage of infection. Based on previous studies on DENV-2 using BALB/c mice as a model for dengue infection (França, Zucoloto & Da Fonseca, 2010; Paes et al., 2005; Paes et al., 2009), liver was among the first organs to be infected and hepatic injury was seen as early as 2 d.p.i. In another study by Paes et al. (2005), hepatic injury in DENV-2 infected BALB/c mice was observed as early as 2 d.p.i, and at the 3 d.p.i, hepatocytes showed diffused steatosis in midzonal areas, while at 7 d.p.i, necrosis and a strong flux of edema was observed. The DENV-1 used in this study was able to evoke inflammatory immune responses as evidenced by the enlargement of spleen and liver (data not shown) and most importantly by the rise of DENV-1 specific IgM and IgG (Wickremsinghe et al., 2018). Here, we also demonstrated that there were differential expressions in the regulating miRNAs during DENV-1 infection.

To date, the miRNA profiling of DENV infections are confined mainly to *in vitro* and mosquito studies (Qi et al., 2013; Campbell et al., 2014; Liu et al., 2015; Liu et al., 2016; Miesen et al., 2016). However, there is scarcity of data on miRNAs differential expression during DENV infection in human (Ouyang et al., 2016; Tambyah et al., 2016). Previous study on DENV-1 infection in human has identified few circulating miRNAs particularly, hsa-miR-21-5p and hsa-miR-146a-5p with high specificity and sensitivity as the promising serum biomarkers for dengue infection (Ouyang et al., 2016). Interestingly, the same miRNAs namely miR-21a-5p and miR-146a-5p were also found in the present study; miR-21a-5p was one of the most abundant miRNAs that was observed in both DENV-1-infected and uninfected control libraries (Fig. 3). However, these miRNAs vary in their expression patterns. hsa-miR-21-5p was upregulated during dengue infection in human (Ouyang et al., 2016), while miR-21a-5p was downregulated by less than 2-fold in DENV-1-infected mouse liver (Fig. 5B). In the present study, miR-146a-5p was upregulated by a 1.23-fold (Data S3), concurring with a previous study reporting an increased expression of miR-146a during DENV infection (Wu et al., 2013); however, hsa-miR-146a-5p was downregulated by at least 3-fold during dengue infection in human (Ouyang et al., 2016). In addition, four of the highly differentially expressed miRNAs found in the present study namely miR-19b-3p, miR-214-3p, miR-340-3p and miR-423-3p were also reported in the study of miRNA expression during dengue infection in human by Tambyah et al. (2016). Yet their expression patterns were in contrast with the study by Tambyah et al. (2016), in which downregulation of miR-19b-3p and upregulation of miR-214-3p, miR-340-3p and miR-423-3p were observed in the present study (Fig. 4). The variation in observation might be due to the type of miRNA analyzed viz. circulating or intracellular miRNA and the host studied viz. human or mouse.

Adaptive immune response is one of the immunopathogenic mechanisms that plays vital roles in major manifestations of dengue (Lei et al., 2001; Whitehorn & Simmons, 2011). In this study, we demonstrated that the TGF-beta, MAPK, PI3K-Akt, Rap1, Wnt and Ras signaling pathways involved in adaptive immune responses were modulated collectively by the high differentially expressed miRNAs during DENV-1 infection in mouse. Three of the pathways are related to each other, in which TGF-beta may induce activation of MAPK signaling pathway and PI3K-Akt signaling pathway. Interestingly, all these six pathways identified in mice liver with dengue, have been shown in human, to play important functional roles of the liver. They have been shown to be closely associated with hepatic inflammatory responses (Li et al., 2016), metabolic dysfunction (Matsuda, Kobayashi & Kitagishi, 2013), liver injury and hepatocarcinogenesis (Behari, 2010; Nakagawa & Maeda, 2012), hepatic fibrosis and chronic liver disease (Blobe, Schiemann & Lodish, 2000; Munshi, Uddin & Glaser, 2011; Shim et al., 2018).

Increases or decreases of TGF-beta have been linked to numerous disease states including atherosclerosis and fibrotic disease of the liver (Blobe, Schiemann & Lodish, 2000). It is well known that liver injury is one of the clinical manifestations associated with dengue infection (Itha et al., 2005; Seneviratne, Malavige & Silva, 2006). Previous study has demonstrated that the development of hepatic fibrosis, a wound-healing response to liver injury, is associated with the pathway mediated by overexpression of cytokine transforming growth factor-beta 1 (TGFB1) (Mawson, 2013; Tanikawa et al., 2017). Moreover, the higher level of TGFB1 in the sera and TGFB1 mRNA in the PBMC has been observed in patients with DHF when compared to DF patients (Agarwal et al., 1999). In another study, plasma obtained from children with DHF from recent DENV-2 outbreaks, were shown to have significantly higher levels of TGFB1 than plasma from children with DF (Laur et al., 1998). Thus, TGFB1 mediated pathway appears likely to play an important role in pathogenesis of liver injury in dengue infection. TGF-beta are multifunctional molecules that regulate processes such as immune function, cell proliferation, differentiation, cell adhesion, haematopoiesis, inflammatory responses and wound healing (Dünker & Krieglstein, 2000). In immune response, TGF-beta acts as a potent immunosuppressor that signals negative regulation in proliferation, differentiation and activation by other cytokines of the TGF-beta secreting immune cells including B-cell, T-cell, macrophages and dendritic cells (Yan, Liu & Chen, 2009). TGF-beta controls adaptive immunity by coordination of development and function of regulatory T cell (Treg) and directs inhibition of cellular activity, and it has also been shown to be linked to depression of innate cells, including natural killer (NK) cells (Wahl, 2007).

Previous studies have shown that DENV induce inflammatory responses involved in liver injury and virus-induced apoptosis via activation of MAPK signaling pathways, thus activation of MAPK signaling pathways is a major cause of liver injury during DENV infection (Sreekanth, Yenchitsomanus & Limjindaporn, 2018). MAPK signaling pathways are activated during DENV infection and the activation of Jun N-terminal kinase (JNK) and p38 MAPK signaling pathways are essential for DENV replication (Ceballos-Olvera et al., 2010). In dengue-infected hepatocyte cells, the activation of JNK, p38, extracellular signal-regulated kinase (ERK) MAPK signaling and Ras signaling pathway induced the overexpression of Regulated on Activation Normal T-cell Expressed and Secreted (RANTES), causing inflammation in the liver (Lee et al., 2008). Furthermore, Wnt signaling has been shown to modulate the type I interferon (IFN) signaling, one of the cellular innate immune pathways, in which the repression on Wnt signaling by miR-34 family induces the activation of type I IFN signaling in response to flavivirus infection including dengue virus and thus inhibiting the viral replication (Smith et al., 2017).

The PI3K-Akt signaling pathway regulates a variety of cellular processes, including cell proliferation, RNA processing, protein translation, autophagy, apoptosis and antiviral immunity (Fulda, 2013). The cellular PI3K-Akt signaling pathway has been shown to play important roles in different steps of the life cycle of viruses. Many DNA and RNA viruses have induced PI3K-Akt signaling pathway for virus survival during infection; these viruses modulate this pathway to optimise the virus entry and replication, virions assembly, latency and reactivation from latency, and apoptosis suppression (Darr, Mauser & Kenney, 2001; Cooray, 2004; Ehrhardt et al., 2006; Saeed et al., 2008; Dunn & Connor, 2011; Yogev & Boshoff, 2013). Lee, Liao & Lin (2005) reported that the activation of PI3K-Akt signaling pathway at the early stage of dengue infection is important in protecting the infected cells from early apoptotic cell death. In this signaling pathway, the expression *Bcl2* gene plays a crucial role in controlling the apoptotic cell death (Lee, Liao & Lin, 2005; Liu et al., 2014). In the present study, the miR-16-5p and miR-182-5p are predicted by microT-CDS to regulate the expression of *Bcl2* gene. Moreover, the target prediction of miR-16-5p was supported with the experimental data. The downregulated expression of miR-16-5p may suppress the cell death via apoptosis by upregulating the expression of *Bcl2* gene, the potent anti-apoptosis factor (Guo et al., 2009; Santosa et al., 2015). Thus, it’s deemed that these two miRNAs are involved in regulating the apoptotic cell death in dengue-infected cells.

An *in silico* study using DenHunt has shown that the dengue viral proteins are interacted directly with some proteins involved in Rap1 signaling pathway (Karyala et al., 2016). Those interactions are believed to interfere with the expression of host miRNA, resulting in a differential expression of some specific miRNAs. Recently, Jiang & Sun (2018) have demonstrated that the Rap1 signaling pathway is modulated by the miRNA targets during DENV-3 infection, which is similar to the findings in this study although the DENV serotype used in our study is different.

## Conclusions

In conclusion, this study demonstrated that the observed highly differentially expressed miRNAs may play vital role in modulating the immune responses during DENV-1 infection *in vivo*. To more clearly understand the roles of each of the highly differentially expressed miRNAs in those signaling pathways during DENV infections, further studies in the characterization of the upstream and downstream proteins involved in both classical and atypical signaling pathways during DENV infection are needed. These studies could potentially identify novel molecular therapies that might modulate the genes of the signaling pathways in DENV infection.

##  Supplemental Information

10.7717/peerj.6697/supp-1 Data S1 Raw and normalized miRNA expression data

10.7717/peerj.6697/supp-2 Data S2 The list of non-detected miRNAs in both libraries

10.7717/peerj.6697/supp-3 Data S3 The normalized differential expression of miRNAs with TPM value more than 10 in both libraries

10.7717/peerj.6697/supp-4 Data S4 The list of non-detected miRNAs in either one of the two libraries

10.7717/peerj.6697/supp-5 Table S1 KEGG pathways of target genes of 31 highly differentially expressed miRNAs in DENV-1-infected mouse liver

## Additional Information and Declarations

### DNA Deposition

The following information was supplied regarding the deposition of DNA sequences: The miRNA sequencing data are available from the NCBI Gene Expression Omnibus (GEO), accession number GSE123346.

### Data Availability

The following information was supplied regarding data availability: The raw data are available in Data S1–S4.

## References

[ref-1] Agarwal R, Elbishbishi EA, Chaturvedi UC, Nagar R, Mustafa AS (1999). Profile of transforming growth factor-beta 1 in patients with dengue haemorrhagic fever. International Journal of Experimental Pathology.

[ref-2] Aye KS, Charngkaew K, Win N, Wai KZ, Moe K, Punyadee N, Thiemmeca S, Suttitheptumrong A, Sukpanichnant S, Prida M, Halstead SB (2014). Pathologic highlights of dengue hemorrhagic fever in 13 autopsy cases from Myanmar. Human Pathology.

[ref-3] Bartel DP (2004). MicroRNAs: genomics, biogenesis, mechanism, and function. Cell.

[ref-4] Behari J (2010). The Wnt/β-catenin signaling pathway in liver biology and disease. Expert Review of Gastroenterology & Hepatology.

[ref-5] Bente DA, Rico-Hesse R (2006). Models of dengue virus infection. Drug Discovery Today: Disease Models.

[ref-6] Blobe GC, Schiemann WP, Lodish HF (2000). Role of transforming growth factor beta in human disease. New England Journal of Medicine.

[ref-7] Burge SW, Daub J, Eberhardt R, Tate J, Barquist L, Nawrocki EP, Eddy SR, Gardner PP, Bateman A (2013). Rfam 11.0: 10 years of RNA families. Nucleic Acids Research.

[ref-8] Campbell CL, Harrison T, Hess AM, Ebel GD (2014). MicroRNA levels are modulated in *Aedes aegypti* after exposure to Dengue-2. Insect Molecular Biology.

[ref-9] Ceballos-Olvera I, Chávez-Salinas S, Medina F, Ludert JE, Angel RMdel (2010). JNK phosphorylation, induced during dengue virus infection, is important for viral infection and requires the presence of cholesterol. Virology.

[ref-10] Chen HC, Lai SY, Sung JM, Lee SH, Lin YC, Wang WK, Chen YC, Kao CL, King CC, Wu-Hsieh BA (2004). Lymphocyte activation and hepatic cellular infiltration in immunocompetent mice infected by dengue virus. Journal of Medical Virology.

[ref-11] Cooray S (2004). The pivotal role of phosphatidylinositol 3-kinase-Akt signal transduction in virus survival. Journal of General Virology.

[ref-12] Darr CD, Mauser A, Kenney S (2001). Epstein-Barr virus immediate-early protein BRLF1 induces the lytic form of viral replication through a mechanism involving phosphatidylinositol-3 kinase activation. Journal of Virology.

[ref-13] Dünker N, Krieglstein K (2000). Targeted mutations of transforming growth factor-beta genes reveal important roles in mouse development and adult homeostasis. European Journal of Biochemistry.

[ref-14] Dunn EF, Connor JH (2011). Dominant inhibition of Akt/protein kinase B signaling by the matrix protein of a negative-strand RNA virus. Journal of Virology.

[ref-15] Ehrhardt C, Marjuki H, Wolff T, Nürnberg B, Planz O, Pleschka S, Ludwig S (2006). Bivalent role of the phosphatidylinositol-3-kinase (PI3K) during influenza virus infection and host cell defence. Cellular Microbiology.

[ref-16] Fabian MR, Sonenberg N, Filipowicz W (2010). Regulation of mRNA translation and stability by microRNAs. Annual Review of Biochemistry.

[ref-17] Fernando SM, Wijewickrama A, Gomes L, Punchihewa C, Madusanka P, Dissanayake H, Jeewandara CK, Peiris H, Ogg G, Malavige N (2016). Factors leading to liver injury in acute dengue infection. International Journal of Infectious Diseases.

[ref-18] França RF, Zucoloto S, Da Fonseca BA (2010). A BALB/c mouse model shows that liver involvement in dengue disease is immune-mediated. Experimental and Molecular Pathology.

[ref-19] Fulda S (2013). Modulation of mitochondrial apoptosis by PI3K inhibitors. Mitochondrion.

[ref-20] Green S, Rothman A (2006). Immunopathological mechanisms in dengue and dengue hemorrhagic fever. Current Opinions in Infectious Diseases.

[ref-21] Guo CJ, Pan Q, Li DG, Sun H, Liu BW (2009). miR-15b and miR-16 are implicated in activation of the rat hepatic stellate cell: an essential role for apoptosis. Journal of Hepatology.

[ref-22] Halstead SB (2002). Dengue hemorrhagic fever: two infections and antibody dependent enhancement, a brief history and personal memoir. Revista Cubana de Medicina Tropical.

[ref-23] Halstead SB (2007). Dengue. The Lancet.

[ref-24] Hierholzer JC, Killington RA, Mahy BWJ, Kangro, HO (1996). Virus isolation and quantitation. Virology methods manual.

[ref-25] Huang KJ, Li SY, Chen SC, Liu HS, Lin YS, Yeh TM, Liu CC, Lei HY (2000). Manifestation of thrombocytopenia in dengue-2-virus-infected mice. Journal of General Virology.

[ref-26] Itha S, Kashyap R, Krishnani N, Saraswat VA, Choudhuri G, Aggarwal R (2005). Profile of liver involvement in dengue virus infection. National Medical Journal of India.

[ref-27] Jiang L, Sun Q (2018). The expression profile of human peripheral blood mononuclear cell miRNA is altered by antibody-dependent enhancement of infection with dengue virus serotype 3. Virology Journal.

[ref-28] Karyala P, Metri R, Bathula C, Yelamanchi SK, Sahoo L, Arjunan S, Sastri NP, Chandra N (2016). DenHunta—comprehensive database of the intricate network of dengue-human interactions. PLOS Neglected Tropical Diseases.

[ref-29] Katagiri K, Hattori M, Minato N, Kinashi T (2002). Rap1 functions as a key regulator of T-cell and antigen-presenting cell interactions and modulates T-cell responses. Molecular and Cellular Biology.

[ref-30] Kozomara A, Griffiths-Jones S (2011). miRBase: integrating microRNA annotation and deep-sequencing data. Nucleic Acids Research.

[ref-31] Kozomara A, Griffiths-Jones S (2014). miRBase: annotating high confidence microRNAs using deep sequencing data. Nucleic Acids Research.

[ref-32] Lapinski PE, King PD (2012). Regulation of Ras signal transduction during T cell development and activation. American Journal of Clinical and Experimental Immunology.

[ref-33] Laur F, Murgue B, Deparis X, Roche C, Cassar O, Chungue E (1998). Plasma levels of tumour necrosis factor alpha and transforming growth factor beta-1 in children with dengue 2 virus infection in French Polynesia. Transactions of the Royal Society of Tropical Medicine and Hygiene.

[ref-34] Lee CJ, Liao CL, Lin YL (2005). Flavivirus activates phosphatidylinositol 3-kinase signaling to block caspase-dependent apoptotic cell death at the early stage of virus infection. Journal of Virology.

[ref-35] Lee YR, Lei HY, Chen SH, Wang JR, Lin YS, Yeh TM, Liu CC, Liu HS (2008). Signaling pathways involved in dengue-2 virus infection induced RANTES overexpression. American Journal of Infectious Diseases.

[ref-36] Lei HY, Yeh TM, Liu HS, Lin YS, Chen SH, Liu CC (2001). Immunopathogenesis of dengue virus infection. Journal of Biomedical Science.

[ref-37] Li CX, Lo CM, Lian Q, Ng KT, Liu XB, Ma YY, Qi X, Yeung OW, Tergaonkar V, Yang XX, Liu H, Liu J, Shao Y, Man K (2016). Repressor and activator protein accelerates hepatic ischemia reperfusion injury by promoting neutrophil inflammatory response. Oncotarget.

[ref-38] Liu YX, Li FX, Liu ZZ, Jia ZR, Zhou YH, Zhang H, Yan H, Zhou XQ, Chen XG (2016). Integrated analysis of miRNAs and transcriptomes in *Aedes albopictus* midgut reveals the differential expression profiles of immune-related genes during dengue virus serotype-2 infection. Insect Science.

[ref-39] Liu Y, Liu H, Zou J, Zhang B, Yuan Z (2014). Dengue virus subgenomic RNA induces apoptosis through the Bcl-2-mediated PI3k/Akt signaling pathway. Virology.

[ref-40] Liu Y, Zhou Y, Wu J, Zheng P, Li Y, Zheng X, Puthiyakunnon S, Tu Z, Chen XG (2015). The expression profile of *Aedes albopictus* miRNAs is altered by dengue virus serotype-2 infection. Cell & Bioscience.

[ref-41] Matsuda S, Kobayashi M, Kitagishi Y (2013). Roles for PI3K/AKT/PTEN pathway in cell signaling of nonalcoholic fatty liver disease. ISRN endocrinology.

[ref-42] Mawson AR (2013). Retinoids, race and the pathogenesis of dengue hemorrhagic fever. Medical Hypotheses.

[ref-43] Miesen P, Ivens A, Buck AH, Van Rij RP (2016). Small RNA profiling in dengue virus 2-infected Aedes mosquito cells reveals viral piRNAs and novel host miRNAs. PLOS Neglected Tropical Diseases.

[ref-44] Mukhopadhyay S, Kuhn RJ, Rossmann MG (2005). A structural perspective of the flavivirus life cycle. Nature Reviews. Microbiology.

[ref-45] Munshi MK, Uddin MN, Glaser SS (2011). The role of the renin-angiotensin system in liver fibrosis. Experimental Biology and Medicine (Maywood).

[ref-46] Murrell S, Wu SC, Butler M (2011). Review of dengue virus and the development of a vaccine. Biotechnology Advances.

[ref-47] Nakagawa H, Maeda S (2012). Molecular mechanisms of liver injury and hepatocarcinogenesis: focusing on the role of stress-activated MAPK. Pathology Research International.

[ref-48] Okkenhaug K, Turner M, Gold MR (2014). PI3K signaling in B cell and T cell biology. Frontiers in Immunology.

[ref-49] Ouyang X, Jiang X, Gu D, Zhang Y, Kong SK, Jiang C, Xie W (2016). Dysregulated serum miRNA profile and promising biomarkers in dengue-infected patients. International Journal of Medical Sciences.

[ref-50] Paes MV, Lenzi HL, Nogueira AC, Nuovo GJ, Pinhão AT, Mota EM, Basílio-de Oliveira CA, Schatzmayr H, Barth OM, Alves AM (2009). Hepatic damage associated with dengue-2 virus replication in liver cells of BALB/c mice. Laboratory Investigation.

[ref-51] Paes MV, Pinhão AT, Barreto DF, Costa SM, Oliveira MP, Nogueira AC, Takiya CM, Farias-Filho JC, Schatzmayr HG, Alves AM, Barth OM (2005). Liver injury and viremia in mice infected with dengue-2 virus. Virology.

[ref-52] Paraskevopoulou MD, Georgakilas G, Kostoulas N, Vlachos IS, Vergoulis T, Reczko M, Filippidis C, Dalamagas T, Hatzigeorgiou AG (2013). DIANA-microT web server v50: service integration into miRNA functional analysis workflows. Nucleic Acids Research.

[ref-53] Pfaffl MW, Horgan GW, Dempfle L (2002). Relative expression software tool (REST©) for group-wise comparison and statistical analysis of relative expression results in real-time PCR. Nucleic Acids Research.

[ref-54] Qi Y, Li Y, Zhang L, Huang J (2013). microRNA expression profiling and bioinformatic analysis of dengue virus-infected peripheral blood mononuclear cells. Molecular Medicine Reports.

[ref-55] Saeed MF, Kolokoltsov AA, Freiberg AN, Holbrook MR, Davey RA (2008). Phosphoinositide-3 kinase-Akt pathway controls cellular entry of Ebola virus. PLOS Pathogens.

[ref-56] Sakinah S, Priya SP, Kumari S, Amira F, Poorani K, Alsaeedy H, Ling MP, Chee HY, Higuchi A, Alarfaj AA, Munusamy MA, Murugan K, Taib CN, Arulselvan P, Rajan M, Neela VK, Hamat RA, Benelli G, Kumar SS (2017). Impact of dengue virus (serotype DENV-2) infection on liver of BALB/c mice: A histopathological analysis. Tissue and Cell.

[ref-57] Samanta J, Sharma V (2015). Dengue and its effects on liver. World Journal of Clinical Cases.

[ref-58] Santosa D, Castoldi M, Paluschinski M, Sommerfeld A, Häussinger D (2015). Hyperosmotic stress activates the expression of members of the miR-15/107 family and induces downregulation of anti-apoptotic genes in rat liver. Scientific Reports.

[ref-59] Seneviratne SL, Malavige GN, Silva HJde (2006). Pathogenesis of liver involvement during dengue viral infections. Transactions of the Royal Society of Tropical Medicine and Hygiene.

[ref-60] Shim KY, Eom YW, Kim MY, Kang SH, Baik SK (2018). Role of the renin-angiotensin system in hepatic fibrosis and portal hypertension. Korean Journal of Internal Medicine.

[ref-61] Smith JL, Jeng S, McWeeney SK, Hirsch AJ (2017). A microRNA screen identifies the Wnt signaling pathway as a regulator of the interferon response during flavivirus infection. Journal of Virology.

[ref-62] Sreekanth GP, Yenchitsomanus PT, Limjindaporn T (2018). Role of mitogen-activated protein kinase signaling in the pathogenesis of dengue virus infection. Cellular Signalling.

[ref-63] Stocks MB, Moxon S, Mapleson D, Woolfenden HC, Mohorianu I, Folkes L, Schwach F, Dalmay T, Moulton V (2012). The UEA sRNA workbench: a suite of tools for analysing and visualizing next generation sequencing microRNA and small RNA datasets. Bioinformatics.

[ref-64] Swafford D, Manicassamy S (2015). Wnt signaling in dendritic cells: its role in regulation of immunity and tolerance. Discovery Medicine.

[ref-65] Tambyah PA, Ching CS, Sepramaniam S, Ali JM, Armugam A, Jeyaseelan K (2016). microRNA expression in blood of dengue patients. Annals of Clinical Biochemistry.

[ref-66] Tanikawa AA, Grotto RM, Silva GF, Ferrasi AC, Sarnighausen VC, Pardini MI (2017). Platelet-derived growth factor A mRNA in platelets is associated with the degree of hepatic fibrosis in chronic hepatitis C. Revista da Sociedade Brasileira de Medicina Tropical.

[ref-67] Tuiskunen A, Wahlström M, Bergström J, Buchy P, Leparc-Goffart I, Lundkvist A (2011). Phenotypic characterization of patient dengue virus isolates in BALB/c mice differentiates dengue fever and dengue hemorrhagic fever from dengue shock syndrome. Virology Journal.

[ref-68] Vlachos IS, Paraskevopoulou MD, Karagkouni D, Georgakilas G, Vergoulis T, Kanellos I, Anastasopoulos IL, Maniou S, Karathanou K, Kalfakakou D, Fevgas A, Dalamagas T, Hatzigeorgiou AG (2015a). DIANA-TarBase v7.0: indexing more than half a million experimentally supported miRNA:mRNA interactions. Nucleic Acids Research.

[ref-69] Vlachos IS, Zagganas K, Paraskevopoulou MD, Georgakilas G, Karagkouni D, Vergoulis T, Dalamagas T, Hatzigeorgiou AG (2015b). DIANA-miRPath v3.0: deciphering microRNA function with experimental support. Nucleic Acids Research.

[ref-70] Wahl SM (2007). Transforming growth factor-beta: innately bipolar. Current Opinion in Immunology.

[ref-71] Whitehorn J, Simmons CP (2011). The pathogenesis of dengue. Vaccine.

[ref-72] Wickremsinghe IAC, Balasubramaniam VRMT, Mot YY, Dhanoa A, Hassan SS (2018). Identification of differentially expressed genes in BALB/c mouse liver upon primary infection with DENV1 and sequential heterologous infection with DENV2. Pathogens.

[ref-73] Wu S, He L, Li Y, Wang T, Feng L, Jiang L, Zhang P, Huang X (2013). miR-146a facilitates replication of dengue virus by dampening interferon induction by targeting TRAF6. Journal of Infection.

[ref-74] Yan X, Liu Z, Chen Y (2009). Regulation of TGF-beta signaling by Smad7. Acta Biochimica et Biophysica Sinica.

[ref-75] Yogev O, Boshoff C (2013). Redefining KSHV latency. Cell Host Microbe.

[ref-76] Zhang YL, Dong C (2005). MAP kinases in immune responses. Cellular & molecular immunology.

