# Supplementary material for: MicroRNA profiling of mouse liver in response to DENV-1 infection by deep sequencing
Source: PeerJ. 2019 Apr 22;7:e6697. doi: 10.7717/peerj.6697 (PMC6482938; doi:10.7717/peerj.6697)
Supplement: Table S1 [file peerj-07-6697-s005.docx]

**Table S1:**

**KEGG pathways of target genes of 31 highly differentially expressed miRNAs in DENV-1-infected mouse liver.**

| **KEGG Pathway** | **Gene Count** | **miRNA Count** | ***p*-Value^*^** |
| --- | --- | --- | --- |
|  |  |  |  |
| ECM-receptor interaction | 35 | 20 | 4.04E-16 |
| Proteoglycans in cancer | 99 | 28 | 1.36E-14 |
| Hippo signaling pathway | 67 | 24 | 7.99E-06 |
| Axon guidance | 64 | 26 | 7.99E-06 |
| FoxO signaling pathway | 63 | 27 | 7.99E-06 |
| PI3K-Akt signaling pathway | 135 | 26 | 3.60E-05 |
| TGF-beta signaling pathway | 39 | 24 | 4.14E-05 |
| Signaling pathways regulating pluripotency of stem cells | 61 | 25 | 4.14E-05 |
| Thyroid hormone signaling pathway | 51 | 24 | 4.82E-05 |
| Fatty acid metabolism | 15 | 12 | 1.58E-04 |
| Fatty acid degradation | 11 | 7 | 1.73E-04 |
| Focal adhesion | 86 | 25 | 2.21E-04 |
| Regulation of actin cytoskeleton | 85 | 26 | 2.29E-04 |
| Pathways in cancer | 145 | 27 | 4.30E-04 |
| Long-term potentiation | 34 | 25 | 7.39E-04 |
| Bacterial invasion of epithelial cells | 35 | 21 | 7.77E-04 |
| Endocytosis | 85 | 26 | 7.77E-04 |
| Adherens junction | 37 | 22 | 8.26E-04 |
| mTOR signaling pathway | 32 | 22 | 9.50E-04 |
| Renal cell carcinoma | 34 | 23 | 1.19E-03 |
| Transcriptional misregulation in cancer | 64 | 25 | 1.19E-03 |
| MAPK signaling pathway | 96 | 27 | 1.19E-03 |
| Estrogen signaling pathway | 38 | 22 | 1.24E-03 |
| Wnt signaling pathway | 60 | 26 | 1.24E-03 |
| Insulin signaling pathway | 60 | 24 | 1.29E-03 |
| Prolactin signaling pathway | 31 | 22 | 2.74E-03 |
| Colorectal cancer | 28 | 23 | 2.74E-03 |
| AMPK signaling pathway | 53 | 22 | 2.78E-03 |
| Glutamatergic synapse | 43 | 24 | 2.78E-03 |
| GABAergic synapse | 29 | 20 | 3.05E-03 |
| Thyroid hormone synthesis | 27 | 18 | 3.90E-03 |
| Acute myeloid leukemia | 26 | 19 | 3.90E-03 |
| Choline metabolism in cancer | 43 | 24 | 3.90E-03 |
| cAMP signaling pathway | 77 | 26 | 3.97E-03 |
| Hedgehog signaling pathway | 25 | 19 | 4.05E-03 |
| cGMP-PKG signaling pathway | 67 | 22 | 4.05E-03 |
| Rap1 signaling pathway | 79 | 25 | 4.05E-03 |
| Melanogenesis | 41 | 23 | 4.24E-03 |
| Neurotrophin signaling pathway | 51 | 26 | 5.63E-03 |
| Inositol phosphate metabolism | 27 | 22 | 7.74E-03 |
| Pancreatic cancer | 29 | 20 | 8.16E-03 |
| Phosphatidylinositol signaling system | 36 | 23 | 9.67E-03 |
| Adrenergic signaling in cardiomyocytes | 56 | 26 | 0.012 |
| Biosynthesis of unsaturated fatty acids | 10 | 11 | 0.014 |
| Dorso-ventral axis formation | 14 | 18 | 0.014 |
| Circadian rhythm | 16 | 20 | 0.014 |
| Basal cell carcinoma | 26 | 20 | 0.014 |
| Prostate cancer | 38 | 22 | 0.015 |
| Long-term depression | 23 | 22 | 0.015 |
| Ras signaling pathway | 77 | 25 | 0.025 |
| Fc gamma R-mediated phagocytosis | 35 | 23 | 0.027 |
| mRNA surveillance pathway | 40 | 26 | 0.027 |
| Oxytocin signaling pathway | 58 | 27 | 0.031 |
| Amoebiasis | 38 | 22 | 0.037 |
| Glioma | 24 | 21 | 0.040 |
| Amphetamine addiction | 27 | 21 | 0.043 |
| ErbB signaling pathway | 34 | 22 | 0.043 |
| Endometrial cancer | 23 | 22 | 0.043 |
| Small cell lung cancer | 33 | 22 | 0.044 |
| Glycosphingolipid biosynthesis - ganglio series | 6 | 10 | 0.047 |
| Arrhythmogenic right ventricular cardiomyopathy (ARVC) | 26 | 23 | 0.048 |
| Glycosaminoglycan biosynthesis - heparan sulfate / heparin | 9 | 11 | 0.049 |
| Hepatitis B | 50 | 24 | 0.049 |
|  |  |  |  |

* Pathway with a *p*-value lower than 0.05 were defined as statistically significant.
